# The two-component system CepRS regulates the cephamycin C biosynthesis in *Streptomyces clavuligerus* F613-1

**DOI:** 10.1186/s13568-019-0844-z

**Published:** 2019-07-27

**Authors:** Jiafang Fu, Ronghuo Qin, Gongli Zong, Chuanqing Zhong, Peipei Zhang, Ni Kang, Xiaoyu Qi, Guangxiang Cao

**Affiliations:** 1 Shandong Medicinal Biotechnology Center, Shandong First Medical University & Shandong Academy of Medical Sciences, Jingshi Road 18877, Jinan, 250062 Shandong People’s Republic of China; 2 Key Laboratory for Biotech-Drugs of National Health Commission, Jinan, 250062 China; 3 School of Municipal and Environmental Engineering, Shandong Jianzhu University, Jinan, 250101 China

**Keywords:** *Streptomyces clavuligerus*, CepRS, Cephamycin C, Clavulanic acid

## Abstract

During industrial fermentation, *Streptomyces clavuligerus* F613-1 simultaneously produces primary product clavulanic acid (CA) and cephamycin C. The cephamycin C biosynthetic gene cluster and pathway have been basically elucidated and the CcaR positive regulator was found to control the cephamycin genes expression. However, additional mechanisms of regulation cannot be excluded. The BB341_RS13780/13785 gene pair in *S. clavuligerus* F613-1 (annotated as SCLAV_2960/2959 in *S. clavuligerus* ATCC27064) encodes a bacterial two-component system (TCS) and were designated as CepRS (for cephamycin regulator/sensor). CepRS significantly affects cephamycin C production but only slightly affects CA production. To further understand the regulation of cephamycin C biosynthesis, the *cepRS* genes were deleted from *S. clavuligerus* F613-1. The deletion mutant resulted in decreased cephamycin C production but had no phenotypic effects. Real-time quantitative polymerase chain reaction analysis revealed that CepRS regulates the expression of most genes involved in cephamycin C biosynthesis, with electrophoretic mobility shift assays showing that CepR interacts with the *cefD*-*cmcI* intergenic region. These results demonstrate that the CepR response regulator serves as a transcriptional activator of cephamycin C biosynthesis, which may provide an approach for metabolic engineering methods for CA production by *S. clavuligerus* F613-1 in future.

**Electronic supplementary material:**

The online version of this article (10.1186/s13568-019-0844-z) contains supplementary material, which is available to authorized users.

## Introduction

The actinomycete *Streptomyces clavuligerus* produces a variety of secondary metabolites, including cephamycin C (7-methoxy-3ʹ-carbamoyl-deacetyl-cephalosporin C) and clavulanic acid (CA). CA is a broad-spectrum inhibitor of β-lactamases produced by penicillin- and cephalosporin-resistant bacteria (Paradkar 2013; Saudagar et al. 2008). *S. clavuligerus* F613-1 is used industrially to produce the β-lactamase inhibitor CA. Cephamycin C, which is also present in the fermentation broth, is considered undesirable when CA is produced (Jin et al. 2015; Qin et al. 2017).

Genes responsible for cephamycin C and CA biosynthesis are clustered in the genome, forming a β-lactam supercluster (Ward and Hodgson 1993). The cephamycin C biosynthetic gene cluster mainly includes ten genes: *pcd*, *lat*, *pcbAB*, *pcbC*, *cefD*, *cefE*, *cmcI, cmcJ*, *cefF*, and *cmcH*. The four genes *pcd*, *lat*, *pcbAB*, *pcbC* are involved in the early stages of cephamycin C biosynthesis. *pcd* and *lat* are involved in the formation of α-aminoadipic. *pcbAB* and *pcbC* are involved in the formation of isopenicillin N. *cefD* and *cefE* are involved in the intermediate stages of cephamycin C biosynthesis, and are responsible for forming deacetylcephalosporin C. The four genes *cmcI, cmcJ*, *cefF*, and *cmcH* are involved in the late stages of cephamycin C biosynthesis and are responsible for the specific C-7 methoxylation and carbamoylation steps (Coque et al. 1995a, b; Enguita et al. 1996).

Secondary metabolites are usually produced at very low levels, indicating the existence of mechanisms that tightly control their biosynthesis (Chater 1993). In fact, the biosynthesis of both cephamycin C and CA is very precisely regulated. Disruption of the biosynthetic or regulatory genes of the cephamycin C/CA supercluster indicated a connection between the biosynthetic pathways of these β-lactam compounds via a regulatory cascade that includes both pleiotropic and pathway-specific regulators (Liras et al. 2008; Martín and Liras 2010). Regulatory gene *ccaR*, located in the cephamycin C biosynthetic gene cluster, encodes protein product CcaR, which appears to be a key *Streptomyces* antibiotic regulatory protein-type positive regulator. Deletion of ccaR completely block the production of both cephamycin C and CA (Pérez-Llarena et al. 1997; Santamarta et al. 2002, 2011). Recently, examination of the DNA-binding characteristics of CcaR revealed that it binds to the *lat*, *cefF*, *cefD*-*cmcI*, and *ccaR* promoter regions in the cephamycin C biosynthetic gene cluster (Kyung et al. 2001; Santamarta et al. 2011). In addition, CcaR affects CA production by binding to the *claR*, *ceaS2* and *oppA1* promoters and, therefore, controlling the transcription of *claR* and consequently of the genes *ceaS2* and *bls2* (Álvarez-Álvarez et al. 2014; Santamarta et al. 2011).

The genome of *S. clavuligerus* F613-1 encodes 47 paired two-component systems (TCSs), amongst which, CagRS has been characterized as a global regulator of both primary and secondary metabolism (Fu et al. 2019). To further study the regulation mechanism of TCSs on the secondary metabolism, we constructed a series of single-gene and double-gene knockout strains. Among them, we found that the biosynthesis of cephamycin C was significantly decreased in the *cepRS* mutant stain, while the biosynthesis of clavulanic acid was slightly affected. The CepRS TCS remains uncharacterized in strain F613-1, although CepR is annotated as the response regulator and CepS as the histidine kinase. The *cepRS* genes are located 1.38 Mb away from the *pcbC* gene of the cephamycin C gene cluster. Sequence analysis indicated that CepS belongs to the sub-family of histidine kinases and CepR belongs to the LuxR family of transcriptional regulators. The LuxR family proteins are approximately 250 amino acids long and include two functional domains: an amino-terminal AHL (*N*-acyl homoserine lactone) binding domain and a carboxy-terminal DNA-binding domain (Nasser and Reverchon 2007). LuxR family response regulators are common in two-component systems (Zhang et al. 2018), however, many of the LuxR family response regulators are orphan response regulators (Fuqua 2006; Patankar and González 2009; Subramoni et al. 2011; Subramoni and Venturi 2009). Here, we show that CepRS affects cephamycin C production and that CepR interacts with the *cefD*-*cmcI* bidirectional promoter. In addition, we found that CepRS has limited effects on CA production. Our results may provide new insights into the regulatory network of the cephamycin C biosynthesis in *S. clavuligerus*.

## Materials and methods

### Bacterial strains and growth conditions

All the strains and plasmids used in this study are listed in Additional file 1: Table S1. Culturing of *Escherichia coli*, *S. clavuligerus* and its derivative strains were performed as described previously (Fu et al. 2019; Qin et al. 2017). In this study, for phenotypic analysis, *S. clavuligerus* F613-1 (CGMCC NO. 12830) and its mutant strains were cultured at 25 °C and 50–60% relative humidity on BSCA (1.5% (w/v) malt extract, 0.3% (w/v) tryptone, 0.4% (w/v) glucose, and 2.0% (w/v) agar powder, pH 7.5) and TSA (3% (w/v) tryptone soya broth, and 2.0% (w/v) agar powder, pH 7.2) plates.

To examine cephamycin C production, strain F613-1 and its derivatives were grown at 25 °C and a relative humidity of 50–60% on TSA plates for 9 days. Agar blocks were collected at days 1, 3, 5, 7, and 9 post-inoculation to determine cephamycin C concentrations. For CA fermentation assays, the culture conditions were performed as described previously (Fu et al. 2019).

### Primers and DNA manipulation

All the primers used in the construction of the Δ*cepRS* mutant and complementation strains, in the confirmation of the transconjugants, and in the electrophoretic mobility shift assays (EMSAs) are listed in Additional file 1: Table S2. The DNA manipulation method were performed as described previously (Fu et al. 2019).

### Construction and complementation of a *cepRS* null mutant and overexpression strain

The *cepRS* genes were knocked out as described previously (Fu et al. 2019). In this study, the upstream homologous arm of the *cepRS* fragment was amplified by PCR using primers *cepRS* L-F/R. The downstream homologous arm of the *cepRS* fragment was PCR-amplified using primers *cepRS* R-F/R. All the recombinant plasmids were verified by DNA sequencing. Conjugation was performed using *S. clavuligerus* F613-1 and *E. coli* ET12567/pUZ8002 as described previously (Fu et al. 2019; Kieser et al. 2000; Sambrook 1989). The Δ*cepRS* mutant strain was confirmed by PCR analysis using the *cepRS* V-F/R primer pair.

For *cepR* gene deletion, the upstream homologous arm of the *cepR* fragment was amplified by PCR using primers *cepRS* L-F/R. The downstream homologous arm of the *cepR* fragment was PCR-amplified using primers *cepR* R-F/R. The mutant strain was confirmed by PCR analysis using the *cepR* V-F/R primer pair. For *cepS* gene deletion, the upstream homologous arm of the *cepS* fragment was amplified by PCR using primers *cepS* L-F/R. The downstream homologous arm of the *cepS* fragment was PCR-amplified using primers *cepRS* R-F/R. The Δ*cepR* and Δ*cepS* mutant strains were confirmed by PCR analysis using the *cepRS* V-F/R primer pair.

For complementation, primers cepRScom-F and cepRScom-R were used to amplify a fragment containing the *cepRS* coding sequence and its native promoter, which was then cloned into *Bam*HI/*Xba*I-digested pSET152, generating recombinant plasmid pSET-cepRS. Plasmids pSET152 and pSET-cepRS were introduced separately into the Δ*cepRS* mutant by conjugation to generate Δ*cepRS*-pSET152 and Δ*cepRS*com strains. The Δ*cepRS*com complemented mutant strain was confirmed by PCR analysis using primer pair *cepRS* V-F/R.

For *cepRS* overexpression, primers cepRS-F/R were used to amplify a fragment containing the *cepRS* coding sequence, which was then cloned into pHLY12, generating recombinant plasmid pHLY-cepRS. Plasmids pHLY-cepRS were introduced into the Δ*cepRS* mutant by conjugation to generate *cepRS* overexpression strain Δ*cepRS-cepRS*. The Δ*cepRS*-*cepRS* complemented mutant strain was confirmed by PCR analysis using primer pair *cepRS* V-F/R.

### Measurement of cell growth and cephamycin C production

Determination of cell growth was performed as described previously (Yu et al. 2012). Briefly, strain F613-1 and the derivative strains were cultured on TSA solid plates covered with cellophane and incubated at 25 °C. For dry weight measurement, cultures were harvested at days 1, 3, 5, 7, and 9 post-inoculation and then dried for 4 h at 80 °C.

Cephamycin C concentrations were determined by agar diffusion bioassay as described previously (Leite et al. 2016). Cephalosporin C zinc salt (J & K Scientific Ltd, CAS #59,143–60-1) was used as a standard, and cephamycin C concentration was measured as “total cephalosporins” present in the sample. *E. coli* ESS 2235, which is highly sensitive to β-lactam antibiotics, was used as the test organism and was cultured for 24 h in LB medium at 37 °C. All samples were treated with penicillinase (Beckton Dickinson, cat. no.: 215,331) to remove penicillin. For biomass measurement and analysis of cephamycin C production, all assays were performed three biological replicates independently. Statistical analysis was analyzed using IBM SPSS Statistics V19.0 software.

## HPLC analysis of CA production

CA concentrations were analyzed by HPLC analysis as previously described (Fu et al. 2019; Jin et al. 2015; Qin et al. 2017). Briefly, the concentration of CA in the SCF fermentation medium was detected by HPLC using an Inertsil ODS-3 4.6 mm × 150 mm, 5 μm column, and using clavulanate lithium as the standard for quantification. All HPLC assays were performed three biological replicates independently. Statistical analysis was analyzed using IBM SPSS Statistics V19.0 software. Biomass was also measured prior to determination of CA production using aliquots (1 g) of fermentation supernatant centrifuged at 6000×*g* to collect mycelia.

## Real-time quantitative polymerase chain reaction (RT-qPCR) analysis

To determine the expression levels of the cephamycin C biosynthesis genes, spores (2 × 10^6^ CFU/ml) from wild-type strain F613-1 and the Δc*epRS* deletion mutant were plated onto TSA medium covered with cellophane and incubated at 25 °C for 72 h. To examine the expression levels of the CA biosynthesis genes, mycelia from *S. clavuligerus* strains F613-1 and Δ*cepRS* were harvested from SCF fermentation broth at 72 h post-inoculation. Total RNA isolation and RT-qPCR procedures were performed as described previously (Fu et al. 2017; Qin et al. 2017). Relative transcript levels were normalized to those of the 16S rRNA gene. For RT-qPCR assays, experiments were performed three biological replicates independently. Statistical analysis was analyzed using IBM SPSS Statistics V19.0 software.

## Expression and purification of His-tagged CepR

*cepR* was amplified using primer pair *cepR*His-F/R (Additional file 1: Table S2) before being cloned into vector pMD18T, generating the intermediate recombinant plasmid pMD18T-cepR. Following DNA sequence confirmation, *cepR* was released from pMD18T-cepR and cloned into the pET-15bto obtain pET-cepR. Finally, pET-cepR was introduced into *E. coli* BL21(DE3). His-tagged CepR expression was induced and the protein was purified as previously described [5, 6]. The purity of His-tagged CepR was determined by 10% SDS-PAGE.

## EMSAs

DNA fragments corresponding to the intergenic regions of the cephamycin C biosynthetic gene cluster were amplified using F613-1genomic DNAas template (primers are listed in Additional file 1: Table S2). EMSAs were performed as previously described (Fu et al. 2019; Zhang et al. 2015).

## Results

### Disruption of *cepRS* leads to a significant reduction in cephamycin C production by *S. clavuligerus* F613-1

CepRS is one of the uncharacterized two-component systems in *S. clavuligerus* F613-1. To investigate the role of the individual components of the CepRS system, each gene was mutated separately in *S. clavuligerus* F613-1. The Δ*cepR* strain, which lacks the coding sequence for the *cepR* response regulator gene, and the Δ*cepS* strain, which lacks the coding sequence for the *cepS* sensor kinase, were constructed (Additional file 1: Figure S1A). Phenotypic differences were not obvious noted for Δ*cepR* or Δ*cepS* compared with F613-1 on BSCA solid medium (Figure S1B). While cephamycin C production was reduced for both Δ*cepR* and Δ*cepS* strains (Additional file 1: Figure S1C), suggesting that CepR and CepS are critical for cephamycin C production.

**Fig. 1:**
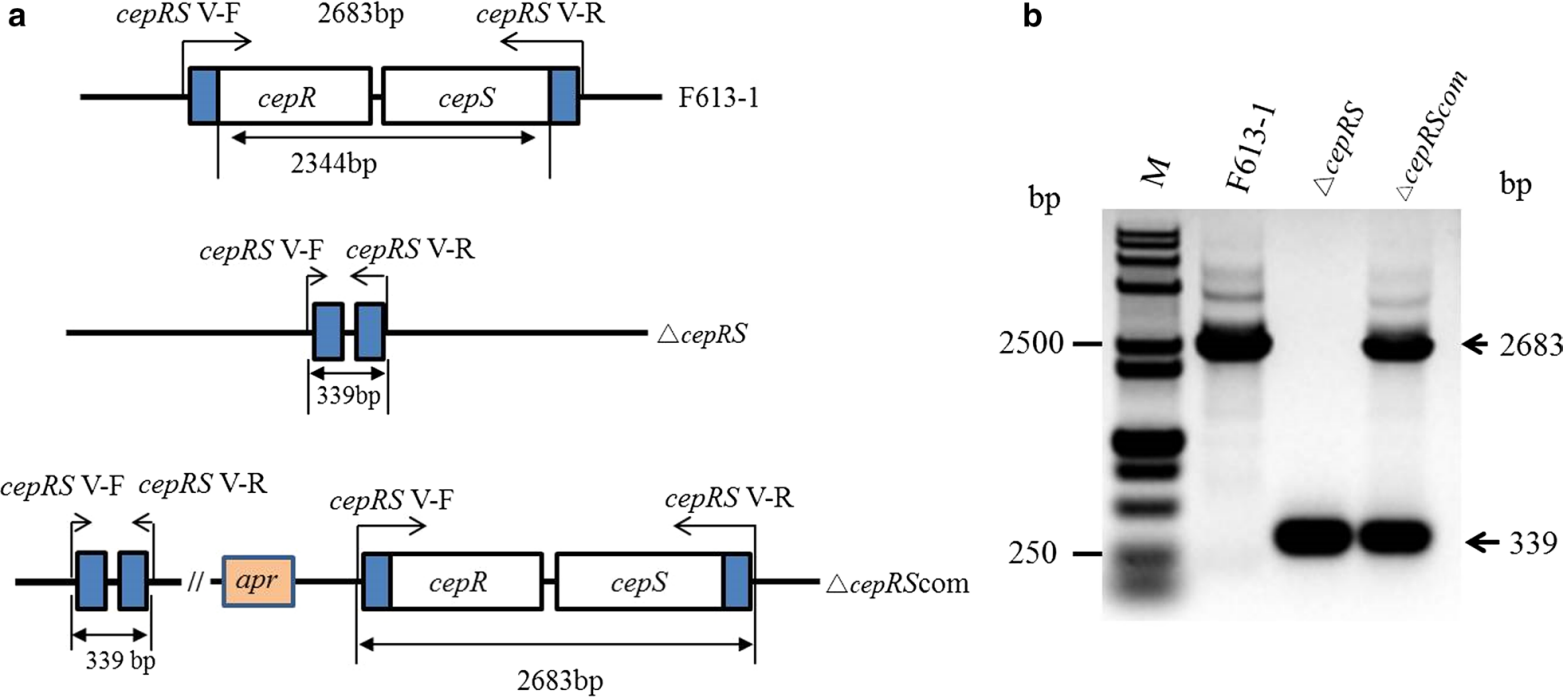
Verification of *cepRS* mutant strain Δ*cepRS*. **a** Schematic representation outlining the construction of the *cepRS* deletion and complementation strains. **b** Polymerase chain reaction-based verification of the Δ*cepRS* and Δ*cepRS*com strains. M: DNA marker

**Fig. 2:**
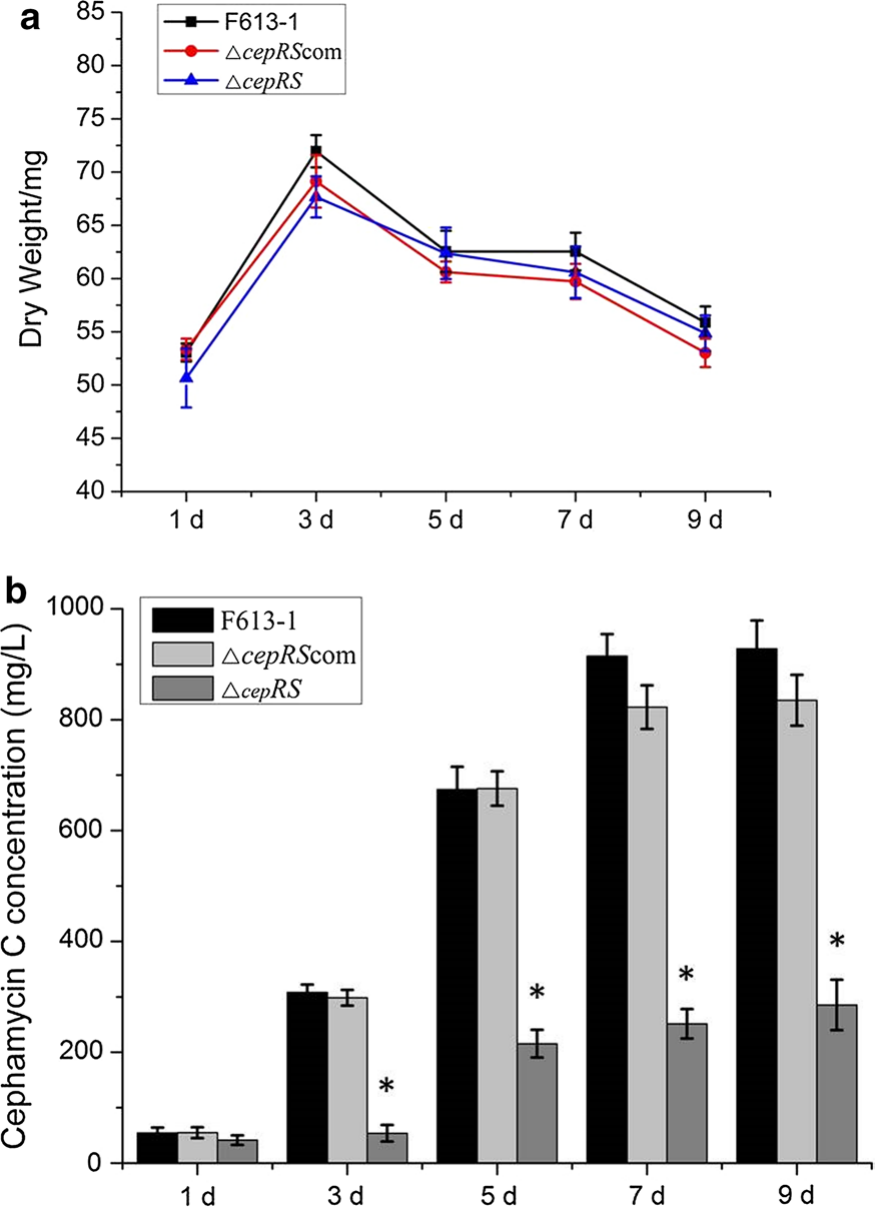
Biomass and cephamycin C concentration analyses of *Streptomyces clavuligerus* strains F613-1, Δ*cepRS*, and Δ*cepRS*com on TSA medium. **a** Growth curves of strains F613-1, Δ*cepRS*, and Δ*cepRS*com grown on TSA plates. Samples for biomass analysis were harvested at days 1, 3, 5, 7, and 9 post-inoculation. Data are the mean ± standard deviation of three separate experiments. **b** Concentrations of cephamycin C produced by *S. clavuligerus* strains F613-1, Δ*cepRS*, and Δ*cepRS*com strains. *Statistically significant difference (P < 0.05) between Δc*epRS* and F613-1 at the same time point

To further characterize the function of CepRS, a *cepRS* null mutant strain, Δ*cepRS*, and a complemented mutant strain, Δ*cepRS*com, were constructed and confirmed by PCR (Fig. 1). No phenotypic differences were noted for Δ*cepRS* relative to parental strain F613-1 on BSCA and TSA solid media (Additional file 1: Figure S1B and data not shown). Interestingly, agar diffusion assays revealed that *cepRS* double gene deletion resulted in a significant reduction of cephamycin C biosynthesis in Δ*cepRS* compared with that of F613-1 despite similar biomass values (Fig. 2). The cephamycin C production was almost restored in the complementation strain Δ*cepRS*com (Fig. 2b). The deletion of *cepRS* was complemented by introduction of *cepRS* genes (Additional file 1: Figure S1A and Fig. 2), suggesting that the CepRS mainly contributes to the regulation of cephamycin C biosynthesis and not affect phenotype of F613-1.

In addition, no phenotypic differences were noted for *cepRS* overexpression strain Δ*cepRS-cepRS* relative to the parental strain F613-1 on BSCA solid media (Additional file 1: Figure S4A). The bioassay data indicated that cephamycin C production was slightly increased in Δ*cepRS*-*cepRS* compared with F613-1 (Additional file 1: Figure S4B).

Over all, the similar phenotypes and decreased cephamycin C production displayed by Δ*cepR*, Δ*cepS*, and Δ*cepRS* support the prediction that CepR and CepS form a paired TCS and indicate that CepRS is important for regulating cephamycin C production.

## *cepRS* regulates the expression of most of the cephamycin C biosynthetic genes

**Fig. 3:**
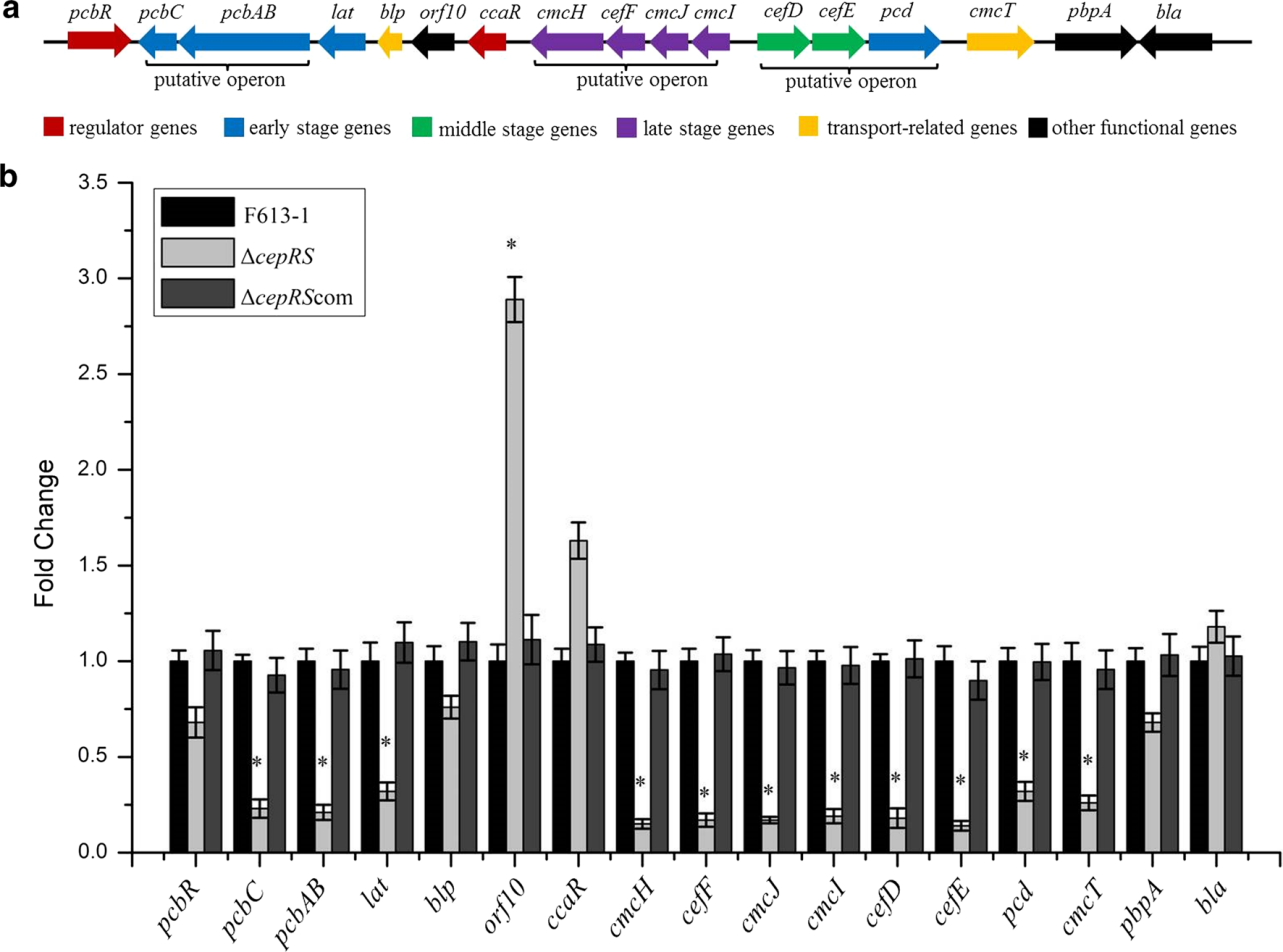
Comparison of gene expression. **a** Schematic diagram of the cephamycin C biosynthetic gene cluster. **b** The expression of genes belonging to the cephamycin C biosynthetic gene cluster was examined by real-time quantitative PCR analysis using RNA extracted from *Streptomyces clavuligerus* strains F613-1, Δ*cepRS* and Δ*cepRS*com. Results were normalized against the expression of the 16S rRNA gene and are shown as fold change over the F613-1 control. Data are the mean ± standard deviation of three independent biological experiments. *Compared with F613-1, P < 0.05

Sequence analysis of the *S. clavuligerus F613-1* genome revealed that this cluster is essentially identical to that of *S. clavuligerus* NRRL27064 (Liras et al. 2008; Cao et al. 2016). As shown in Fig. 3a, three putative operons (*pcbAB*-*pcbC*, *cmcI-cmcJ-cefF-cmcH*, and *cefD-cefE-pcd*) were predicted in the gene cluster based on intervals between the ORFs. Because the CepRS TCS appears to significantly affect cephamycin C production, RT-qPCR analysis was performed to gain further insights into the mechanisms of CepRS regulation of cephamycin C biosynthesis. As shown in Fig. 3b, the expression of *pcbR*, *blp*, *ccaR*, *pbpA*, and *bla* was not significantly affected by disruption of CepRS; however, the expression of uncharacterized gene *orf10* was significantly increased in Δ*cepRS* compared with that in F613-1. Further, the expression levels of all of the biosynthetic genes *pcbC*, *pcbAB*, *lat*, *cefD*, *cefE*, *cmcI*, *cmcJ*, *cefF*, and *cmcH* were significantly reduced in the Δ*cepRS* mutant compared with those in F613-1. The RT-qPCR data revealed that expression of genes in the early (*pcbC*, *pcbAB* and *lat*), middle (*cefD* and *cefE*), and late (*cmcI*, *cmcJ*, *cefF*, *cmcH*) stages of cephamycin C biosynthesis was reduced in Δ*cepRS* compared with F613-1, which is consistent with the agar diffusion assay results showing that cephamycin C levels were reduced in the Δ*cepRS* mutant compared with wild-type strain F613-1. In addition, the RT-qPCR data revealed that the effect on expression level of cephamycin C biosynthetic genes was not significantly affected by *cepRS* overexpression compared with F613-1 (Additional file 1: Figure S4C). Overall, the above data suggest that CepRS positively regulates the expression of most of the cephamycin C biosynthetic genes.

## CepR specifically binds to the promoters of *cefD-cmcI*

**Fig. 4:**
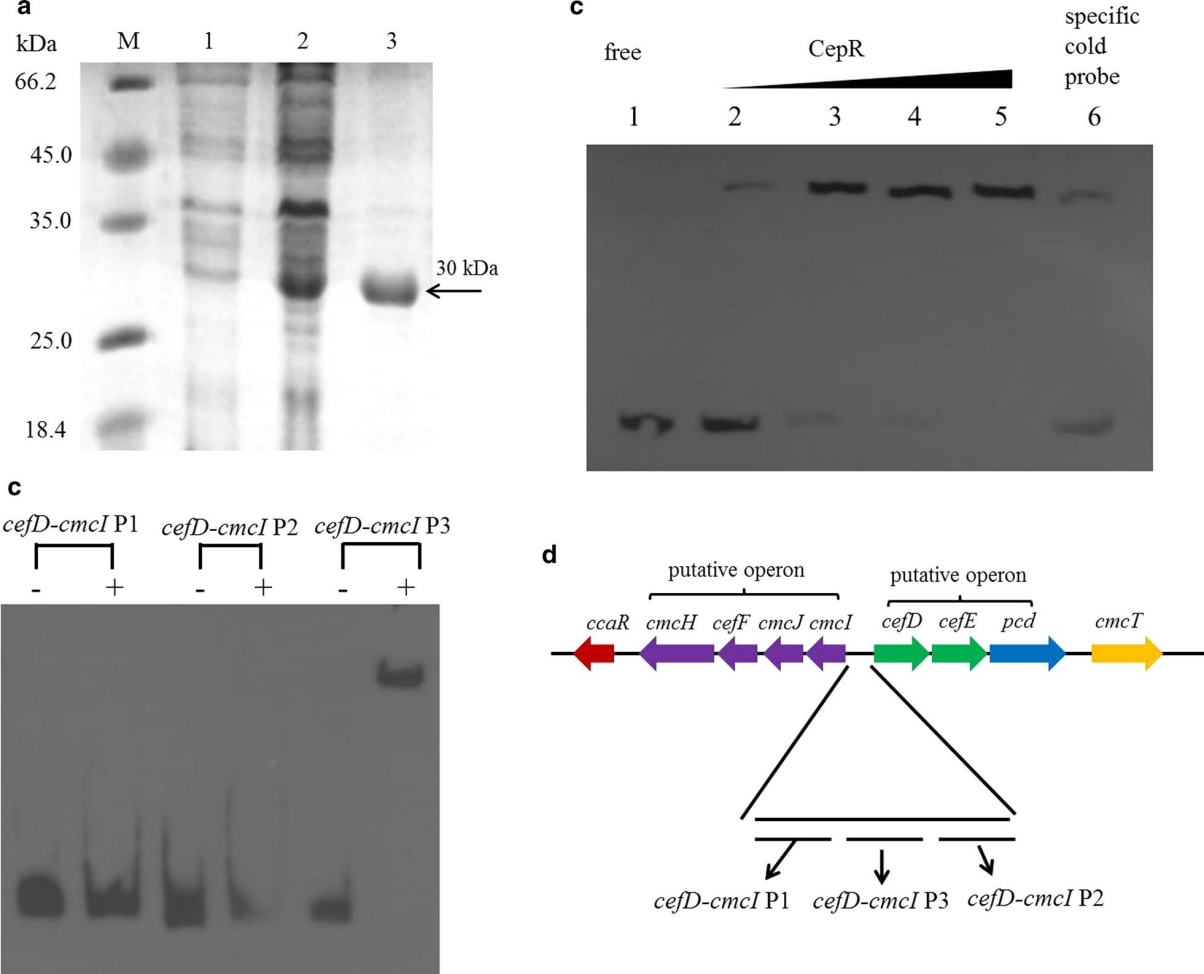
Binding of CepR to the *cefD*-*cmcI* promoter regions. **a** Expression and purification of His-tagged CepR analyzed by sodium dodecyl sulfate polyacrylamide gel electrophoresis. Lane 1, control sample. Lane 2, His-tagged CepR induced by the addition of IPTG. Lane 3, purified fusion protein. Lane M, protein marker. **b** Electrophoretic mobility shift assay analysis of the binding of His-tagged CepR to the promoter regions of *cefD*-*cmcI*. The P1, P2, and P3 *cefD*-*cmcI* intergenic regions were 208 bp, 315 bp, and 318 bp, respectively. The probes were incubated either with no protein ( − ) or with 3.0 μg of CepR ( + ).1.0 µg polydI/dC was used as a competitor. **c** A fixed amount of probe P3 was incubated with no CepR (Lane 1), 0.5–3.0 µg of CepR (Lanes 2–5), or 3.0 μg of CepR and 100-fold excess of unlabeled specific probe (Lane 6). 1.0 µg polydI/dC was used as a competitor. **d** Positions of P1–P3 *cefD*-*cmcI* intergenic regions in the cephamycin C biosynthetic gene cluster

The above data indicated that the CepRS TCS affects the expression of 12 genes in the cephamycin C biosynthetic gene cluster. Therefore, we speculated that response regulator CepR may interact directly with the promoters of the affected genes. To investigate the targets of CepR, we amplified from the intergenic regions upstream of the genes different DNA fragments to use them as probes in EMSAs: 196 bp upstream region of *pcbAB*, two upstream regions (330 bp and 313 bp respectively) of *lat*, two upstream regions (148 bp and 193 bp respectively) of *orf10*, two upstream regions (117 bp and 208 bp respectively) of *ccaR*, three intergenic regions (208 bp, 315 bp and 318 bp respectively) of *cefD-cmcI*, 149 bp upstream region of *cmcT*, 210 bp upstream region of *pbpA*. Recombinant 6 × His-CepR was expressed and purified for EMSAs (Fig. 4a). When incubated with the purified His-tagged CepR, obvious shifting was only observed with probe containing intergenic region P3 of *cefD-cmcI* (Fig. 4b), indicating that CepR interacts with *cefD-cmcI* intergenic region P3. However, shifting was not observed with probes containing the intergenic regions of other genes in the cephamycin C biosynthetic gene cluster. Further EMSA experiments showed that shifting was almost abrogated by the addition of excess specific unlabeled probe P3 (Additional file 1: Fig. 4c, lanes 2–6), indicating that binding of CepR to the *cefD-cmcI* P3 intergenic region was specific. These in vitro results suggested that CepR interacts directly with the promoters of two critical genes (*cefD* and *cmcI*) necessary for cephamycin C biosynthesis. However, the *cefD-cmcI* intergenic region lies between the *cmcI-cmcJ-cefF-cmcH* and *cefD-cefE-pcd* operons (Fig. 4d), indicating that response regulator CepR may directly regulate the expression of all seven genes (*cmcI*, *cmcJ*, *cefF*, *cmcH*, *cefD*, *cefE*, and *pcd*).

## Discussion

### CepRS affects cephamycin C production but not cell growth

A bacterial TCS consists of a sensor histidine kinase and a response regulator (Fu et al. 2019). TCSs mediate adaptation to changing environments and are reported to be involved in a variety of bacterial cellular responses, including morphological development, biofilm development, sporulation, osmoregulation, chemotaxis, photosynthesis, antibiotic production, and pathogenicity (Bijlsma and Groisman 2003; Fu et al. 2019; Hutchings et al. 2004; Mikkelsen et al. 2011; Ogura and Tanaka 2002). TCSs are highly abundant in *Streptomyces* species and reportedly affect the production of antibiotics such as CA (Fu et al. 2019). Here, we presented a typical and paired TCS (CepRS) in *S. clavuligerus*, bioinformatics analysis revealed that CepS is a histidine kinase containing histidine phosphotransfer domain and ATPase domain, while CepR is a response regulator containing phosphorylation domain and DNA binding domain, function analysis showed that mutation of *cepRS*, or each gene individually, leads to a significant reduction in cephamycin C production but does not affect phenotype or biomass. Δ*cepS*, Δ*cepR*, and the double gene deletion mutant Δ*cepRS* has similar effect on the cephamycin C production, indicating CepRS is a paired TCS and works together. We further determined that CepRS regulates the expression of multiple genes critical for cephamycin C biosynthesis, most notably *pcbC*, *pcbAB*, *lat*, *cmcH*, *cefF*, *cmcJ*, *cmcI*, *cefD*, *cefE*, *pcd*, and *cmcT.* Therefore, CepRS may be a specific TCS for the regulation of cephamycin C biosynthesis, while the functions of many other TCSs remain unclear.

## Possible mechanisms for CepRS regulation of cephamycin C biosynthesis

In the cephamycin C biosynthetic gene cluster of *S. clavuligerus*, a strong bidirectional promoter located between *cefD* and *cmcI* could give rise to two polycistronic transcripts: *cefD*-*cefE*-*pcd* and *cmcI*-*cmcJ*-*cefF*-*cmcH* (Santamarta et al. 2011). Our RT-qPCR data revealed that the *cefD*-*cefE*-*pcd* and *cmcI*-*cmcJ*-*cefF*-*cmcH* genes in *S. clavuligerus* strain F613-1 likely form two polycistronic transcripts because the expression levels of the grouped genes are almost identical (Fig. 3). In addition, RT-qPCR data also revealed that CepR negatively regulated the resistance gene *orf10*. The *orf10* expression level was affected by CcaR (Santamarta et al. 2011), also affected by pimM (Martínez-Burgo et al. 2019), the presence of regulators CcaR, PimM and CepR imply that the biosynthesis of cephamycin C is intricately regulated.

Additionally, the *cmcI*-*cefD* bidirectional promoter was characterized as a target of CepR in *S. clavuligerus* F613-1 (Fig. 4). Therefore, CepR may simultaneously control the expression of early- (*pcd*), middle- (*cefD*, *cefE*), and late-stage (*cmcI*, *cmcJ*, *cefF*, *cmcH*) genes of the cephamycin C biosynthetic pathway. In addition, CcaR reportedly also binds to the *cmcI*-*cefD* bidirectional promoter (Santamarta et al. 2002); however, our RT-qPCR data revealed that CepRS does not affect *ccaR* transcription, indicating that CepRS and CcaR may independently regulate cephamycin C production. Further, our RT-qPCR data revealed that expression of *pcbC*, *pcbAB*, and *lat* was also significantly reduced in Δ*cepRS* compared with that in F613-1. CepR could not bind to the intergenic fragments used in the EMSA assays of the three genes: *pcbC*, *pcbAB*, and *lat*; however, a binding sequence located upstream of probes used in this work cannot be excluded. It has been reported that the response regulator AfsR binds to *afsS* after phosphorylation in *Streptomyces coelicolor* (Lee et al. 2002). But we found that the response regulator CepR did not bind to promoter region of *cepS* when we analyzed the binding targets of CepR in EMSA.

The pathway specific regulator CcaR belongs to the family of SARP proteins and positively regulate the production of cephamycin C (Pérez-Llarena et al. 1997; Santamarta et al. 2011, 2002). CcaR were reported to be bind to the *cefD-cmcI* intergenic region (Santamarta et al. 2011). Like CcaR, CepR also positively regulate the production of cephamycin C and could interact with *cefD-cmcI* intergenic region (P3). A direct repeat sequence GGCGGTCGATCGGCGGT in P3 region was found by hand, which might be a CepR box, while the significance of these sequences will remain unclear until footprinting experiments confirm their binding to CepR. In addition, the presence of two positive regulators CcaR and CepR suggesting that the biosynthesis of cephamycin C has a complex regulation mechanism.

## CepRS does not affect clavulanic acid biosynthesis

Because genes involved in the biosynthesis of cephamycin C and CA form a supercluster (Ward and Hodgson 1993), we also examined whether CepRS affects CA production in *S. clavuligerus* F613-1. HPLC was carried out to analyze the concentration of CA in Δ*cepRS* and F613-1 in SCF fermentation medium (Additional file 1: Figure S2). HPLC results revealed that there are no obvious differences in CA concentrations between strains Δ*cepRS* and F613-1 (Additional file 1: Figure S2). There are three clusters of genes involved in CA biosynthesis in *S. clavuligerus*: the CA biosynthetic gene cluster, the clavam gene cluster, and the paralogue gene cluster (Fu et al. 2019). RT-qPCR-based analysis of the effects of CepRS on the expression of the CA biosynthetic genes failed to identify any significant changes in the expression of these genes in the Δ*cepRS* mutant compared with wild-type strain F613-1 (Additional file 1: Figure S3). The RT-qPCR data are also consistent with HPLC results showing no obvious differences in CA concentrations between strains Δ*cepRS* and F613-1. Therefore, these findings suggest that CepRS mainly regulates the production of cephamycin C and has little effect on CA production. As such, it may be possible to increase CA production by modifying CepRS, providing an approach for metabolic engineering efforts for CA production by *S. clavuligerus* F613-1 in future.

## Additional files

**Additional file 1: Table S1.** Plasmids and strains used in this study. **Table S2.** Primers used in this study. **Figure S1.** (A) Polymerase chain reaction-based verification of the Δ*cepS* and Δ*cepR* strains. M: DNA marker. (B) Phenotype of F613-1, △*cepRS*, △*cepR*, △*cepS* and △*cepRS* on BSCA solid media. (C) Ceph C (cephamycin C) obtained in F613-1, △*cepRS*, △*cepR* and △*cepS* strains on TSA solid medium. *, statistically significant difference (P < 0.05) between △*cepRS*, △*cepR*, △*cepS* and F613-1 at the same time point. **Figure S2.** Biomass and clavulanic acid (CA) concentration of F613-1, △*cepRS* and the complemented strain. A: Growth curves of F613-1, △*cepRS* and △*cepRS*com in SCF fermentation medium. Samples for growth curve analysis were harvested at five time points (1, 3, 5, 7 and 9 d). Data are the mean ± SD of three independent experiments. B: Analysis of the change of CA concentration during fermentation. Data are the mean ± SD of three independent biological experiments. **Figure S3.** Expression of genes in the CA biosynthetic gene cluster were examined by RT-qPCR between F613-1 (black bars), △*cepRS* (grey bars) and △*cepRS*com (dark grey bars). Results were normalized for 16S rRNA gene content and are shown as fold change over the F613-1 control. Data are the mean ± SD of three independent biological experiments. **Figure S4**. A: Phenotype of F613-1, △*cepRS*, △*cepRS*com and △*cepRS*-*cepRS* (over-expression strain) on BSCA solid media. B: Cephamycin C obtained in F613-1, △*cepRS* and △*cepRS-cepRS* (over-expression strain) strains on TSA solid medium. *, compared with F 613-1, P < 0.05. Data are the mean ± SD of three independent biological experiments. C: Expression of genes in the cephamycin C biosynthetic gene cluster were examined by RT-qPCR between F613-1 (black bars), △*cepRS* (grey bars) and △*cepRS*-*cepRS* (over-expression strain) (dark grey bars). Results were normalized for 16S rRNA gene content and are shown as fold change over the F613-1 control. *, compared with F613-1, P < 0.05. Data are the mean ± SD of three independent biological experiments.

## Data Availability

Please contact author for data requests.

## Abbreviations

TCS: two-component system
CepRS: cephamycin regulator/sensor
CA: clavulanic acid
EMSA: electrophoretic mobility shift assay
HPLC: high performance liquid chromatography
RT-qPCR: real-time quantitative polymerase chain reaction
