# Supplementary material for: The two-component system CepRS regulates the cephamycin C biosynthesis in Streptomyces clavuligerus F613-1
Source: AMB Express. 2019 Jul 27;9:118. doi: 10.1186/s13568-019-0844-z (PMC6661058; doi:10.1186/s13568-019-0844-z)
Supplement: Supplementary file 1 — Additional file 1: Table S1. Plasmids and strains used in this study. Table S2. Primers used in this study. Figure S1. (A) Polymerase chain reaction-based verification of the ΔcepS and ΔcepR strains. M: DNA marker. (B) Phenotype of F613-1, △cepRS, △cepR, △cepS and △cepRS on BSCA solid media. (C) Ceph C (cephamycin C) obtained in F613-1, △cepRS, △cepR and △cepS strains on TSA solid medium. *, statistically significant difference (P < 0.05) between △cepRS, △cepR, △cepS and F613-1 at the same time point. Figure S2. Biomass and clavulanic acid (CA) concentration of F613-1, △cepRS and the complemented strain. A: Growth curves of F613-1, △cepRS and △cepRScom in SCF fermentation medium. Samples for growth curve analysis were harvested at five time points (1, 3, 5, 7 and 9 d). Data are the mean ± SD of three independent experiments. B: Analysis of the change of CA concentration during fermentation. Data are the mean ± SD of three independent biological experiments. Figure S3. Expression of genes in the CA biosynthetic gene cluster were examined by RT-qPCR between F613-1 (black bars), △cepRS (grey bars) and △cepRScom (dark grey bars). Results were normalized for 16S rRNA gene content and are shown as fold change over the F613-1 control. Data are the mean ± SD of three independent biological experiments. Figure S4. A: Phenotype of F613-1, △cepRS, △cepRScom and △cepRS-cepRS (over-expression strain) on BSCA solid media. B: Cephamycin C obtained in F613-1, △cepRS and △cepRS-cepRS (over-expression strain) strains on TSA solid medium. *, compared with F 613-1, P < 0.05. Data are the mean ± SD of three independent biological experiments. C: Expression of genes in the cephamycin C biosynthetic gene cluster were examined by RT-qPCR between F613-1 (black bars), △cepRS (grey bars) and △cepRS-cepRS (over-expression strain) (dark grey bars). Results were normalized for 16S rRNA gene content and are shown as fold change over the F613-1 control. *, compared with F613-1, P < 0.05. Data are [file 13568_2019_844_MOESM1_ESM.pdf]

Additional file 1

Journal name: AMB Express

Article title: The two-component system CepRS regulates the cephamycin C biosynthesis in *Streptomyces clavuligerus* F613-1

Author names: Jiafang Fu[1, 2†], Ronghuo Qin[1†], Gongli Zong[1, 2], Chuanqing Zhong[3], Peipei Zhang[1, 2], Ni Kang[1], Xiaoyu Qi[3], Guangxiang Cao[1, 2]*

[1] Shandong Medicinal Biotechnology Center, Shandong First Medical University & Shandong Academy of Medical Sciences, Jinan 250062, China

[2] Key Laboratory for Biotech-Drugs of National Health Commission, Jinan 250062, China

[3] School of Municipal and Environmental Engineering, Shandong Jianzhu University, Jinan 250101, China

[†] Jiafang Fu and Ronghuo Qin have contributed equally.

*Corresponding author: Guangxiang Cao, caozhong0402@163.com

Tel.: +86 531 8291 9606; Fax: +86 531 8291 9645

Shandong Medicinal Biotechnology Center,

Shandong First Medical University & Shandong Academy of Medical Sciences,

Jingshi Road 18877, Jinan 250062, Shandong, China.

Captions

1. Table S1 Plasmids and strains used in this study.

2. Table S2 Primers used in this study.

3. Figure S1. (A) Polymerase chain reaction-based verification of the Δ*cepS* and Δ*cepR* strains. M: DNA marker. (B) Phenotype of F613-1, △*cepRS*, △*cepR*, △*cepS* and △*cepRS* on BSCA solid media. (C) Ceph C (cephamycin C) obtained in F613-1, △*cepRS*, △*cepR* and △*cepS* strains on TSA solid medium. *, statistically significant difference (P < 0.05) between △*cepRS*, △*cepR*, △*cepS* and F613-1 at the same time point.

4. Figure S2. Biomass and clavulanic acid (CA) concentration of F613-1, △*cepRS* and the complemented strain. A: Growth curves of F613-1, △*cepRS* and △*cepRS*com in SCF fermentation medium. Samples for growth curve analysis were harvested at five time points (1, 3, 5, 7 and 9 d). Data are the mean ± SD of three independent experiments. B: Analysis of the change of CA concentration during fermentation. Data are the mean ± SD of three independent biological experiments.

5. Figure S3. Expression of genes in the CA biosynthetic gene cluster were examined by RT-qPCR between F613-1 (black bars), △*cepRS* (grey bars) and △*cepRS*com (dark grey bars). Results were normalized for 16S rRNA gene content and are shown as fold change over the F613-1 control. Data are the mean ± SD of three independent biological experiments.

6. Figure S4. A: Phenotype of F613-1, △*cepRS*, △*cepRS*com and △*cepRS-cepRS* (over-expression strain) on BSCA solid media. B: Cephamycin C obtained in F613-1, △*cepRS* and △*cepRS-cepRS* (over-expression strain) strains on TSA solid medium. *, compared with F 613-1, P < 0.05. Data are the mean ± SD of three independent biological experiments. C: Expression of genes in the cephamycin C biosynthetic gene cluster were examined by RT-qPCR between F613-1 (black bars), △*cepRS* (grey bars) and △*cepRS-cepRS* (over-expression strain) (dark grey bars). Results were normalized for 16S rRNA gene content and are shown as fold change over the F613-1 control. *, compared with F613-1, P < 0.05. Data are the mean ± SD of three independent biological experiments.

Table S1 Plasmids and strains used in this study.

Strains or plasmids	Description	Source/Reference
***Streptomyces clavuligerus* strains**		
F613-1	Industrial clavulanic acid producer	(Qin 2017)
△*cepRS*	F613-1 mutant with *cepRS* double-gene deletion	This study
△*cepR*	F613-1 mutant with *cepR* gene deletion	This study
△*cepS*	F613-1 mutant with *cepS* gene deletion	This study
△*cepRScom*	△*cepRS* complemented with *cepRS*	This study
Δ*cepRS-cepRS*	△*cepRS* overexpressed with *cepRS*	This study
△*cepRScom*-pSET152	△*cepRS* complemented with the empty vector pSET152	This study
***E. coli* strains**		
DH5α	General cloning host	Trans (China)
BL21 (DE3)	Strain used for protein expression	Trans (China)
E. coli ESS 2235	a supersensitive organism to beta-lactam antibiotics	(Leite et al. 2016)
ET12567/pUZ8002	Strain used for conjugation between *E. coli* and *Streptomyces spp*	(Kieser et al. 2000)
**Plasmids**		
pJTU1278	*E. coli-Streptomyces* shuttle vector, *tsr*[a] *bla*[a] *oriT*	(He et al. 2010)
pHLY12	*Streptomyces* overexpression vector with the strong promoter ermEp	Preserved in our lab
pHLY-cepRS	pHLY12 containing the coding sequence of *cepRS*	This study
pSET152	*Streptomyces* integrated vector	BioVector (China)
pET-15b	Expression vector containing the T7 promoter and 6×His-thrombin	Novagene (USA)
pSET-cepRS	pSET152 containing the coding sequence of *cepRS* plus *cepR* upstream intergenic sequence	This study
pET-cepR	pET15b containing the coding sequence of *cepR*	This study
pEasy-Blunt-Simple	General cloning vector	Trans (China)
pJTU-cepR	pJTU1278 containing the *cepR*-disrupted cassette	This study
pJTU-cepS	pJTU1278 containing the *cepS*-disrupted cassette	This study
pJTU-cepRS	pJTU1278 containing the *cepRS*-disrupted cassette	This study

[a] *tsr*, thiostrepton-resistance gene; *bla*, ampicillin-resistance gene.

Table S2 Primers used in this study.

**Oligonucleotides**	**DNA Sequence (5'→3')***
**Recombinant plasmid pJTU-cepRS, pJTU-cepS, pJTU-cepR construction**	
cepRS L-F	GGACTAGTACCACATAGGAACGGGTA
cepRS L-R	CCTCTAGAAATGCAAGCTTGTGAAGACCTATGTGAGCC
cepRS R-F	GGAAGCTTGATCTCCACGAGGATCATGC
cepRS R-R	CGTCTAGAGGAAACAGCCGGAAACAG
cepR R-F	GGACTAGTAGTCACCCTCACTCAGCC
cepR R-R	CCTCTAGAAATGCAAGCTTTGCACGCCCTTCAGGAGC
cepS L-F	GGACTAGTTTCCCCCACTGCTTCGGC
cepS L-R	CCTCTAGAAATGCAAGCTTAGGTGGAGGGCTGATCCG
**Validation of △*cepRS*, △*cepS*, △*cepR* and △*cepRS*com strain**	
cepRS V-F	CTGCACCCGGTTCTCGCA
cepRS V-R	GTGAAGCTCTCATCCTCGA
**Recombinant plasmid pSET-cepRS construction**	
cepRScom-F	GGCTGCAGGTCGACTCTAGACCTCGTCGTCCGCCCCGCTC
cepRScom-R	TCGCGCGCGGCCGCGGATCCCCATGCATCGCCGGGGTCCA
Recombinant plasmid pHLY-cepRS construction	
cepRS-F	GGAATTCCATATGCGGATCGGCGCCCCCGTCAT
cepRS-R	CTAGTCTAGACCGCCGAGACCGGGGTGTGA
**Recombinant plasmid pET-cepR construction**	
*cepR*His-F	CATATGACGGGGGCGCCGATCCGGGTGGTCATCGCC
*cepR*His-R	CTCGAGTCAGCCCACGCCCAGACCCGCGTCCCGCGC
**EMSAs**	
cmcI-cefD p1 For	TCGTTCATTGCCCTCTTCCTTGAGTG
cmcI-cefD p1 Rev	ATGGTCGTCCGATCGCCGCA
cmcI-cefD p2 For	CGCCGTTGTGCACCCATGGG
cmcI-cefD p2 Rev	CCCAGTCGGCTACCGCCATGTC
cmcI-cefD p3 For	TGCGGCGATCGGACGACCAT
cmcI-cefD p3 Rev	CCCATGGGTGCACAACGGCG
lat p For1	GCCCATGGGTGAGAACTCCTGGG
lat p Rev1	CGGTCCCAGGCTTCGATGGC
lat p For2	GCCATCGAAGCCTGGGACCG
lat p Rev2	CAACTGCCCTGAAGCGGGCC
ccaR For1	TGGCTTCGGCGTAATCCTTG

ccaR Rev1	CTGTCCCAAATCGTCCATGC
ccaR For2	TCGGTGAACCCGGAAGAACC
ccaR Rev2	TTTGCCGAGGATTTCCGGAC
orf10 For1	TTTGAGTACCGTCCGCCGCC
orf10 Rev1	TTCCTCACACAGAGCAGACC
orf10 For2	GGTCTGCTCTGTGTGAGGAA
orf10 Rev2	GTTTCCCTGAACCAACGCTG
pcbAB For	CATCATTCGTGGGCTCTCCG
pcbAB Rev	TGGCGAGCAGTGTCACGGCG
cmcT For	GCATGCCGCACGGATGACGC
cmcT Rev	CAGCGGAACTCCCTCGCATG
pbpA For	TACCAGCGGAAGGAGGCACC
pbpA Rev	CTGTCTCCTTCATACGCCGC
**RT-qPCR**	
16S-RT For	GAGATCCGCCTTCGCCACCG
16S-RT Rev	CTGCATTCGATACGGGCAGGC
pcbR-F	CCAACCTCAAGCCGACGAAG
pcbR-R	ACGCGACCTTCCACTCCTTG
pcbC-F	TGACCGACCAGGAGAAGCAC
pcbC-R	GACCGCCTTGTAGTAGCCGT
pcbAB-F	GCCTATCTCACCTACACCTC
pcbAB-R	AGCGTCTGCCCGTTGATGAG
lat-F	AGGCACTTGAGCAGCATATG
lat-R	ATGCTGGGCGGGTTGATTCC
blp-F	ATGGTGAAGAAGACATGGAG
blp-R	TGGTCGGCACCGGCGAGCTG
orf10-F	CCGATGTCCCGCAGTTGTTG
orf10-R	CCTGGATGACGTCGTCGATC
ccaR-F	TTCGCGGATGTGACCTCCAG
ccaR-R	TCATCAGGCTCACATACAGG
cmcH-F	GTTCTTCACCTCCACGCACG
cmcH-R	CCGAAGAGGAAGGAGTAGCG
cefF-F	TCTTCAACCTCGCCGCACTG
cefF-R	AAGAAGTCCATCGCCGTGTC
cmcJ-F	ACGCGGTGGAGTTCTTCGAC

cmcJ-R	TCGACGTGCACCCGCAGATG
cmcI-F	CCCACGCCAACACCTTCAAC
cmcI-R	CGATGATGAAGTAGTCGCCC
cefD-F	CCACCGTCGTCAACCTCAAC
cefD-R	AGCAGGAAGTCCATCGGCTC
cefE-F	CACCCTCATCCAGCAGACAC
cefE-R	TGCCCGCTATCTGGTCCCTG
pcd-F	TCACCGAGCACAAACAGGAC
pcd-R	TCGCAGATGTCGATCATCTC
cmcT-F	AGGTGCCGCTCTGGTACTTC
cmcT-R	AGCGAGACCAGCAGCATGAC
pbp74-F	AGAGGGGTCGGAAAAGGCTG
Pbp74-R	GCTCCGGCTTCGGCTCCTTG
bla-F	TCGCCTTCTGCTCCACGTTC
bla-R	GGTGAGATCGAGTCGACGTC
gcas-RT For	CACCCCTGGCCGACTATGCC
gcas-RT Rev	GCCCGTGGGTGTACCAGGAC
orf16-RT For	CACCGTCTGCTTCCCGCACG
orf16-RT Rev	GCGGTGCTTGGTCATGTCGG
oppA2-RT For	ACGTCTGGGTGTGGCTGCTC
oppA2-RT Rev	GCAGCCGGTAGGTCCAGGTC
orf14-RT For	CGGCGAACGACGACGAAACG
orf14-RT Rev	CCAGTCGTCGAGGGCGGTC
orf13-RT For	TCCTCTCCGCGATGCGGTTC
orf13-RT Rev	GCATGCCGATGTCGATGGCG
orf12-RT For	AGGGCCGACAAGGAGCGATG
orf12-RT Rev	GTCCGGACGAGGTCAGCAGC
fd-RT For	GCCCCCGAGATCTTCGACCAG
fd-RT Rev	TAGCCCTCGGTGACCGTGAT
cyp450-RT For	AGCCAGGTGTGGCTGGTGAC
cyp450-RT Rev	GCGGATGAACGACGCCGACT
cad-RT For	CCGACTGGACCCGGATGATCG
cad-RT Rev	TTCGTGGCCTGGTAGACGGC
claR-RT For	TGCTGTCGCTGGTCTCCACG
claR-RT Rev	TAGGCCGCGTCCACCTGGTA

oppA2-RT For	ACGTCTGGGTGTGGCTGCTC
oppA2-RT Rev	GCAGCCGGTAGGTCCAGGTC
oat2-RT For	CGACTTCACCGTCCTCGCCT
oat2-RT Rev	GGTCGCGACATTCGCGTTGC
cas2-RT For	CTCCGAGCTTCCCGAGGTGC
cas2-RT Rev	CGCGCAGCAGCAGATAACCG
pah2-RT For	ACGGCGCAGAGCCATCTGTC
pah2-RT Rev	TTGGTGTCGGAGTGCGCGTC
bls2-RT For	TGCCGCTGTACACCTGTGTGG
bls2-RT Rev	CGCGGGCACCTGGTAGACAC
ceaS2-RT For	AGGCCGCGTCGATTCTCTTCG
ceaS2-RT Rev	AGAGGTTGGTCATACCGGGGC
cas1-RT For	GAGGACCGCTCCCTGCTGAC
cas1-RT Rev	CTCCGAGGACAGGTGGTGCG

* Restriction enzyme sites are underlined and were used for cloning purposes.

Figure S1

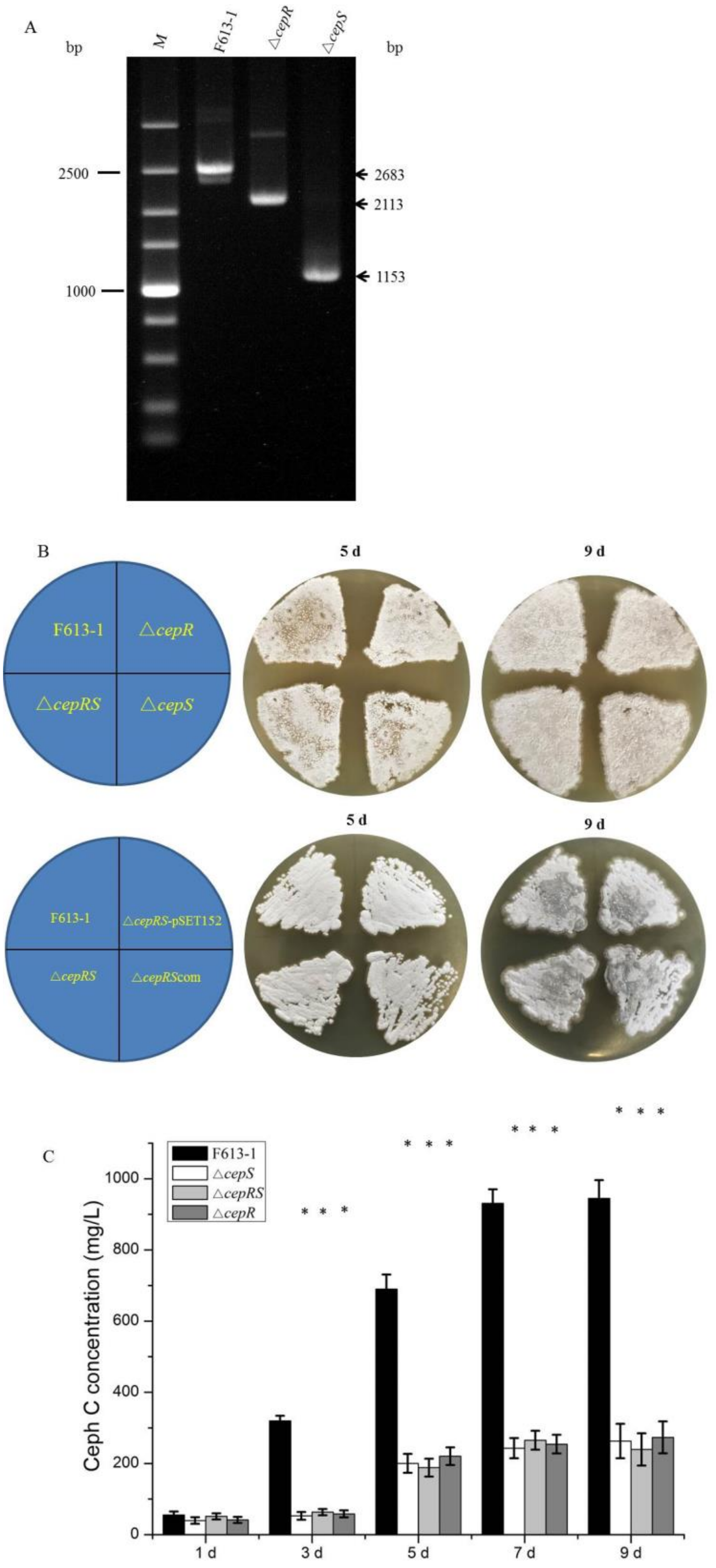

Figure S1. (A) Polymerase chain reaction-based verification of the Δ*cæpS* and Δ*cæpR* strains. M: DNA marker. (B) Phenotype of F613-1, △*cepRS*, △*cepR*, △*cepS* and △*cepRS* on BSCA solid media. (C) Ceph C (cephamycin C) obtained in F613-1, △*cæpRS*, △*cæpR* and △*cæpS* strains on TSA solid medium. *, statistically significant difference ($P < 0.05$) between △*cepRS*, △*cepR*, △*cepS* and F613-1 at the same time point.

Figure S2

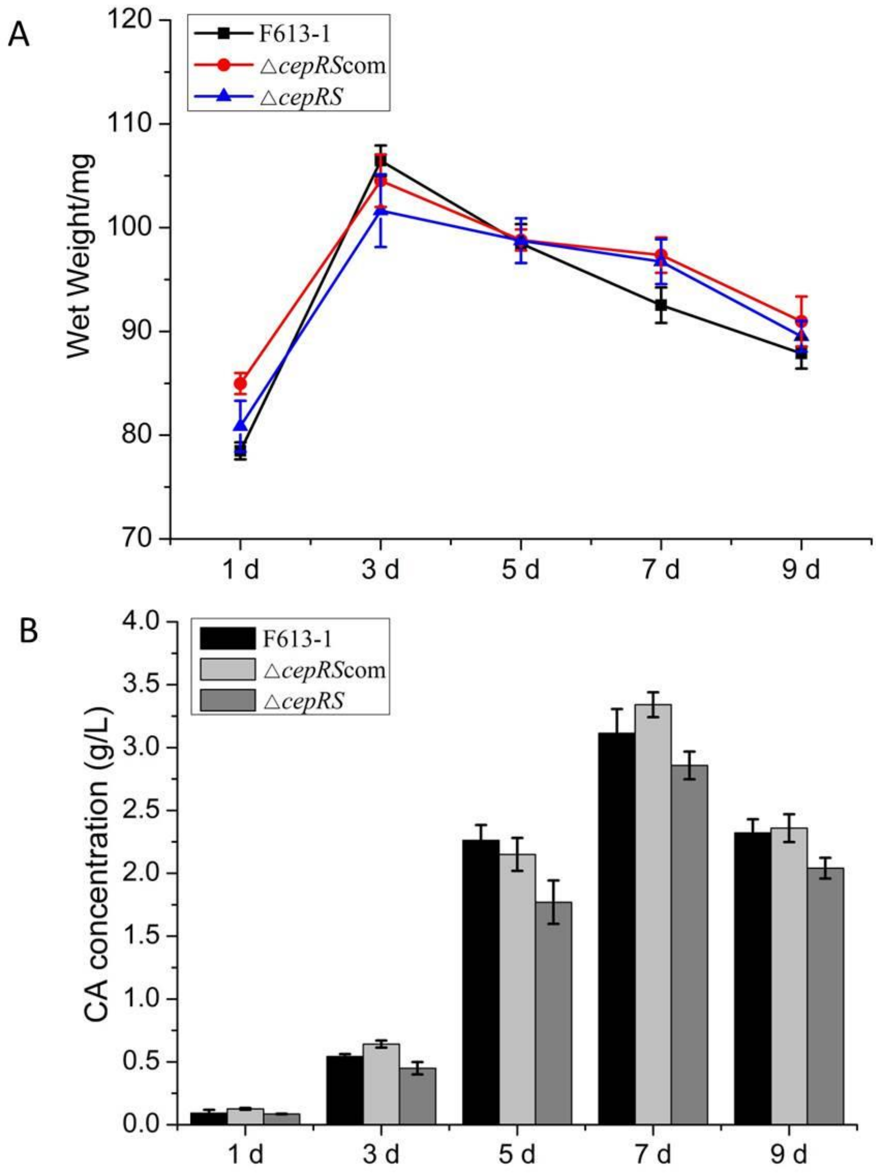

Figure S2. Biomass and clavulanic acid (CA) concentration of F613-1, △*cepRS* and the complemented strain. A: Growth curves of F613-1, △*cepRS* and △*cepRS*com in SCF fermentation medium. Samples for growth curve analysis were harvested at five time points (1, 3, 5, 7 and 9 d). Data are the mean ± SD of three independent experiments. B: Analysis of the change of CA concentration during fermentation. Data are the mean ± SD of three independent biological experiments.

Figure S3

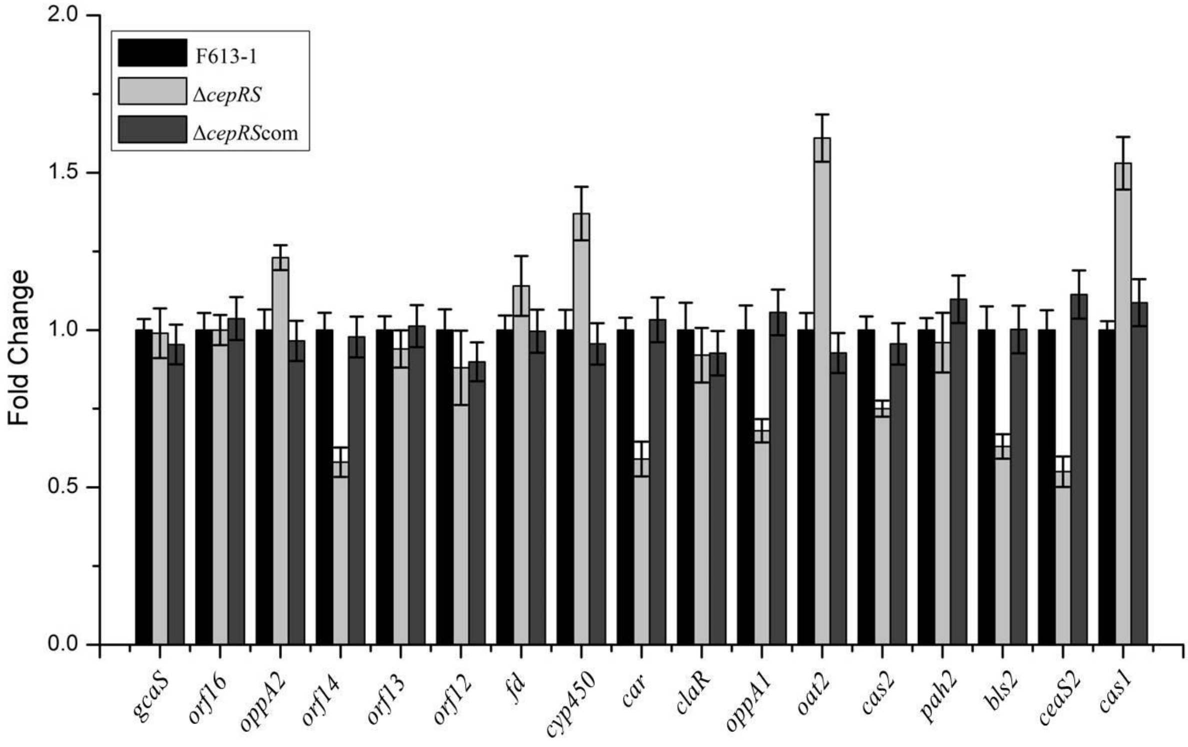

Figure S3. Expression of genes in the CA biosynthetic gene cluster were examined by RT-qPCR between F613-1 (black bars), △*cepRS* (grey bars) and △*cepRS*com (dark grey bars). Results were normalized for 16S rRNA gene content and are shown as fold change over the F613-1 control. Data are the mean ± SD of three independent biological experiments.

Figure S4

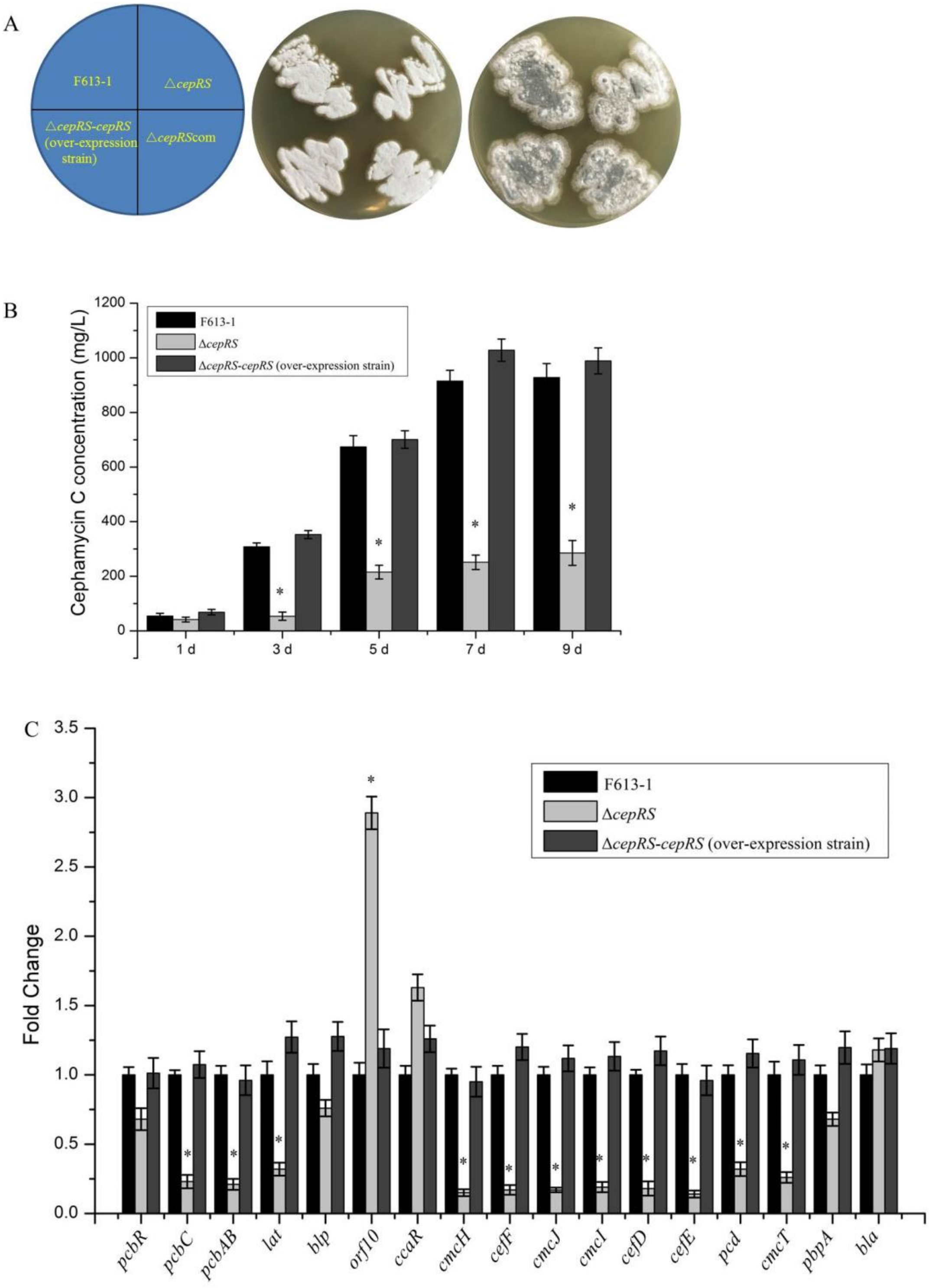

Figure S4. A: Phenotype of F613-1, △*cepRS*, △*cepRS*com and △*cepRS-cepRS* (over-expression strain) on BSCA solid media. B: Cephamycin C obtained in F613-1, △*cepRS* and △*cepRS-cepRS* (over-expression strain) strains on TSA solid medium. *, compared with F 613-1, $P < 0.05$. Data are the mean ± SD of three independent biological experiments. C: Expression of genes in the cephamycin C biosynthetic gene cluster were examined by RT-qPCR between F613-1 (black bars), △*cepRS* (grey bars) and △*cepRS-cepRS* (over-expression strain) (dark grey bars). Results were normalized for 16S rRNA gene content and are shown as fold change over the F613-1 control. *, compared with F613-1, $P < 0.05$. Data are the mean ± SD of three independent biological experiments.

References:

He Y, Wang Z, Bai L, Liang J, Zhou X, Deng Z (2010) Two pHZ1358-derivative vectors for efficient gene knockout in *streptomyces*. Journal of microbiology and biotechnology 20(4):678-82

Kieser T, Bibb MJ, Buttner MJ, Chater KF, Hopwood DA (2000) Practical *Streptomyces* Genetics. John Innes Foundation, Norwich, England

Leite CA, Cavallieri AP, Baptista AS, Araujo ML (2016) Dissociation of cephamycin C and clavulanic acid biosynthesis by 1,3-diaminopropane in *Streptomyces clavuligerus*. FEMS microbiology letters 363(1):fnv215

Qin R, Zhong, C., Zong, G., Fu, J., Pang, X., Cao, G. (2017) Improvement of clavulanic acid production in *Streptomyces clavuligerus* F613-1 by using a claR - neo reporter strategy. Electronic Journal of Biotechnology 28:41-46
